# Editorial: Pharmaceuticals, personal care products and endocrine disrupting chemicals: The physiological consequences of exposure to pollutants in aquatic animals

**DOI:** 10.3389/fphys.2023.1145052

**Published:** 2023-01-30

**Authors:** Mingzhe Yuan, Caterina Faggio, Monia Perugini, Valbona Aliko, Youji Wang

**Affiliations:** ^1^ Department of Biomedical Sciences and Centre of Reproduction, Development and Aging (CRDA), Faculty of Health Sciences, University of Macau, Taipa, Macau, China; ^2^ Department of Chemical, Biological, Pharmaceutical, and Environmental Sciences, University of Messina, Messina, Italy; ^3^ Department of Bioscience and Technology for Food, Agriculture, and Environment, University of Teramo, Teramo, Italy; ^4^ Department of Biology, Faculty of Natural Sciences, University of Tirana, Tirana, Albania; ^5^ International Research Center for Marine Biosciences at Shanghai Ocean University, Ministry of Science and Technology, Shanghai, China

**Keywords:** pharmaceuticals and personal care products (PPCPs), endocrine disrupting chemicals (EDCs), aquatic organisms, water pollution, physiological effects

Water pollution is considered as one of the biggest environmental and public health problems worldwide. The main cause of the contamination is artificial chemicals. The contaminants in the environment mostly originate from industry, agriculture, hospital, and domestic wastewater (Rock et al., 2009; Chilke, 2018). Therefore, aquatic animals will inevitably be exposed to contamination through the polluted habitat. With exposure to contamination comes to a high risk of biomagnification, i.e., the accumulation of toxic substances in living organisms increases as one rises to the next trophic level, along the food chain, so that the concentration of pollutants is greater in the predator than that the prey (Drouillard, 2008). Water pollution is particularly difficult to deal with because aquatic organisms are captive to lifetime, even multigenerational exposure. The accumulation of pollutants in the tissues of organisms then exerts some adverse effects.

Among these contaminants, pharmaceuticals and personal care products (PPCPs), such as contraceptives, medicines, perfumes, makeup, and toothpastes (Liu & Wong, 2013; Faggio et al., 2016; Ebele et al., 2017; Cahova et al., 2021; Zicarelli et al., 2022), along with the endocrine disrupting chemicals (EDCs), which can be found in different products including pesticides, children’s products, food contact materials, electronics, and building materials, medical tubing, antibacterial products, textiles and clothing (Lauretta et al., 2019; Kahn et al., 2020), are the two major classes of chemicals that are commonly found in the aquatic environment. Besides the general toxicity, EDCs and some PPCPs can interfere with the body endocrine system by acting as the “hormone mimics” which can activate or block the specific downstream signaling pathways of some hormones. This interference eventually results in adverse effects such as lowering of the immune defenses, hormonal imbalances, and other dysfunctions that compromise the survival of aquatic organisms and the balance of the whole ecosystem (Ebele et al., 2017; Kahn et al., 2020). In “*Pharmaceuticals, Personal Care products and Endocrine Disrupting Chemicals: the physiological consequences of exposure to pollutants in aquatic animals*,” we provide a set of five original research articles to reveal the physiological consequences from the exposure to both PPCPs and EDCs in aquatic animals and lay a basis to understand the toxic effects of environmentally persistent pollutants.

Pharmaceuticals and personal care products are considered as emerging contaminants, and their presence in aquatic system induces the risk to the marine ecosystem, as they may adversely affect non-target organisms that are exposed to them. In this context, the work by Pagano et al. evaluated the time-dose effects of anti-inflammatory drugs, acetylsalicylic acid (ASA), on the mussel *Mytilus galloprovincialis*. They found that chronic exposure to ASA caused physiological changes in *M. galloprovincialis* by regulating the cell volume in the digestive gland and inducing inflammation from a histological level. In addition, a time-dose reaction to ASA in the gills and digestive gland showing numerous alterations such as lipofuscin deposits and hemocyte infiltration was found. In the study of peracetic acid (PAA), an oxidative disinfectant with a broad spectrum of antimicrobial activity, Carletto et al. demonstrated that low-dose PAA (1 mg/L) did not pose a significant threat to salmon parr health in a freshwater recirculating aquaculture system (RAS) with pulse treatment. However, in the continuous treatment group, the changes of the molecular and phenotypic indicators (i.e., expression of antioxidant genes, 8-oxo-2′-deoxyguanosine and o,o’-dityrosine) collectively reveal that PAA could trigger stress in the skin, gills, liver, and dorsal fins, and especially the mucosa. In addition, this consequence was not chronic.

EDCs are a group of chemicals that alter the endocrine system causing adverse effects on the health of aquatic animals (Zhou et al., 2019; Aliko et al., 2022; Merola et al., 2022; Tresnakova et al., 2023). The concerning risk of the most of EDCs are estrogenic effects, which can result in feminization, hermaphroditism, and sex reversion that may reduce the reproductive capacity of populations and increase extinction risk (Tohyama et al., 2015). In this context, Chen et al. compared effects of three EDCs (17-α-ethinylestradiol (EE2), Bisphenol A (BPA) and Non-ylphenol (NP)) on the expression of estrogen receptors at mRNA and protein level in the white cloud mountain minnow *Tanichthys albonubes*. The results showed that EE2, BPA, and NP treatment all upregulated the expression of ERα, ERβ1, and ERβ2 in the brain, liver, and testis with dose-dependent effect. As for the effects of EDCs on the reproductive system, the work by Sayed et al. demonstrated that 4-non-ylphenol (4-NP) treatment could disrupt the expression of sex hormone [17β-estradiol (E2) and testosterone (T)] and the alteration of histopathological, hematological and biochemical indicators in gonad tissues were found after 4-NP treatment. In addition, 15-day recovery period was insufficient to remove the negative effects caused by 4-NP. Except the classic EDCs, some novel synthesized chemicals may also have endocrine disrupting effects. Zhang et al. demonstrated that metamifop (MET), a novel aryloxyphenoxy propionate herbicide, increased vitellogenin (VTG) levels in liver and plasma in the female rice field eels *Monopterus albus*. In addition, MET exposure increased the expression of CYP19A1b and CYP17 that regulate sex hormone production in the brain, but the expression of genes (CYP19A1a, CYP17, FSHR, LHCGR, hsd11b2, 3β-HSD) associated with sex hormone secretion in the ovary and the estrogen receptor genes (esr1, esr2a, esr2b) in the liver were all suppressed. In addition, the expression of sex-related gene (Dmrt1) was suppressed.

In summary, the articles in this Research Topic presented recent advances in the field of toxicity, endocrine disrupting and physiological consequences from the time-dose exposure to both PPCPs and EDCs in aquatic animals. The works by Carletto et al. and Sayed et al. also revealed carry-over effects of the contaminants after recovery phase. In addition, Zhang et al. investigated a relative new EDC, MET, and its effects on the fish reproduction. These papers provide some novel research insights and directions for the future research on using the aquatic animals to understand the potential ecological risks of PPCPs and EDCs.
